# Supplementary figures and images for: A detailed kinetic model of Eastern equine encephalitis virus replication in a susceptible host cell
Source: PLoS Comput Biol. 2025 Jun 4;21(6):e1013082. doi: 10.1371/journal.pcbi.1013082 (PMC12136344; doi:10.1371/journal.pcbi.1013082)

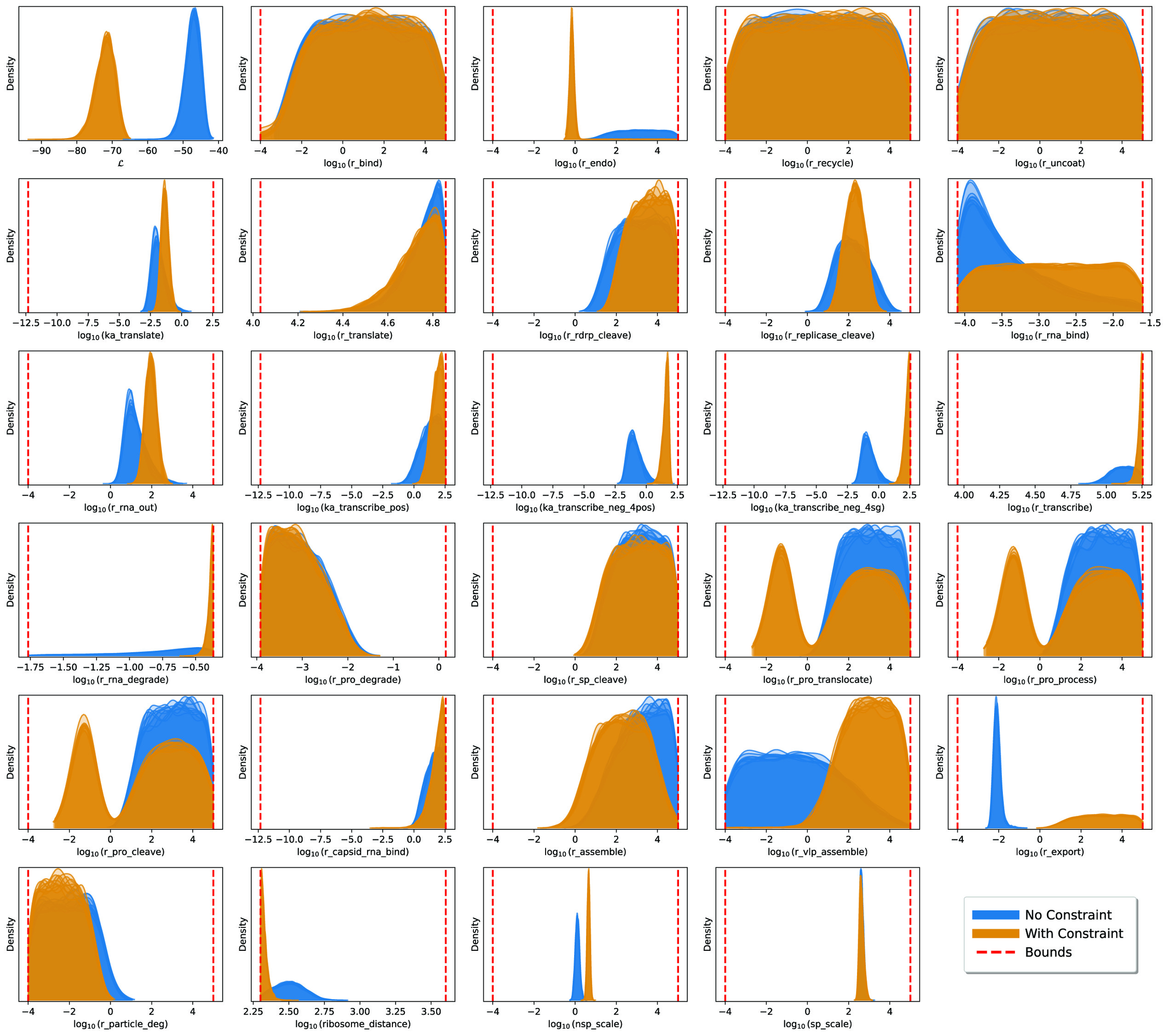

Supplement: S1 Fig — Top left plot shows likelihood distribution as calculated by Eq 11. The remaining plots show 26 of the 36 model parameters in the BioNetGen model are calibrated as well as 2 scaling factors, nsp_scale and sp_scale, which are used to scale nonstructural and structural polyprotein concentrations to luminescence. These distributions are from the posterior ensemble of 2,500 parameter sets after calibrating the model given different set of initial particles. Specifically, the model was calibrated 10 times with the RNA genome strand constraint (blue) and 10 times without the constraint (orange). Vertical, dashed red lines indicate the parameter bounds provided during model calibration. (TIF) [file pcbi.1013082.s003.tif]

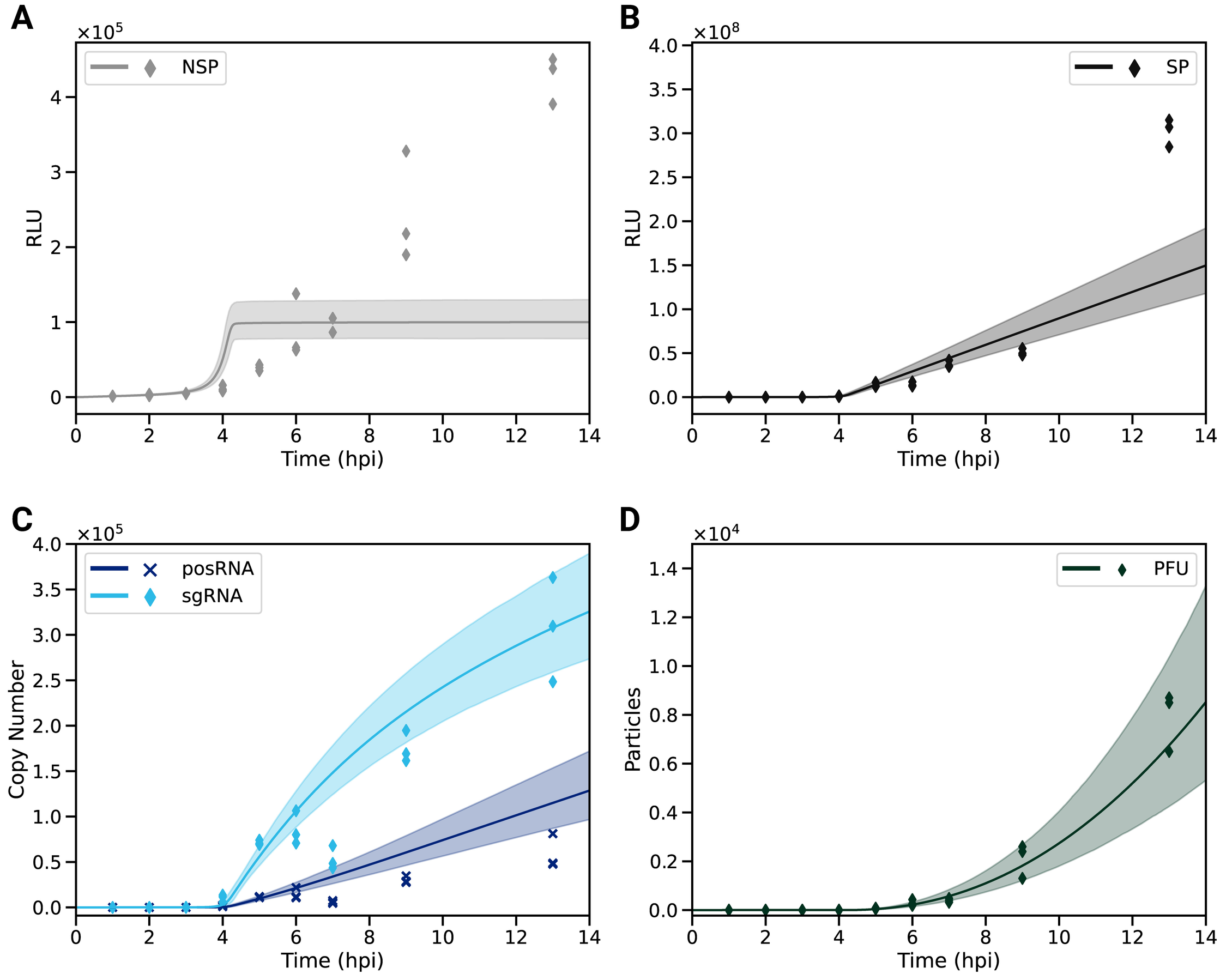

Supplement: S2 Fig — The posterior samples from model calibration (n=2500) trajectory weighted means (line) and 95th percentiles (shaded) are shown in all panels. The mean and standard deviation of experimental data (n = 3 replicates) are plotted from the protein luminescence assays, RT-qPCR, and plaque assay (see Materials and methods). Fits to protein luminescence data of nonstructural protein 3 (nsP) and structural protein (sP) in relative light units (RLUs) are shown in panels (A) and (B), respectively. (C) RNA dynamics of the positive-sense genome (posRNA, dark blue) and subgenomic RNA (sgRNA, light blue) are shown. (D) Model trajectories of viral particle production of plaque-forming, infectious particles (PFU). (TIF) [file pcbi.1013082.s004.tif]

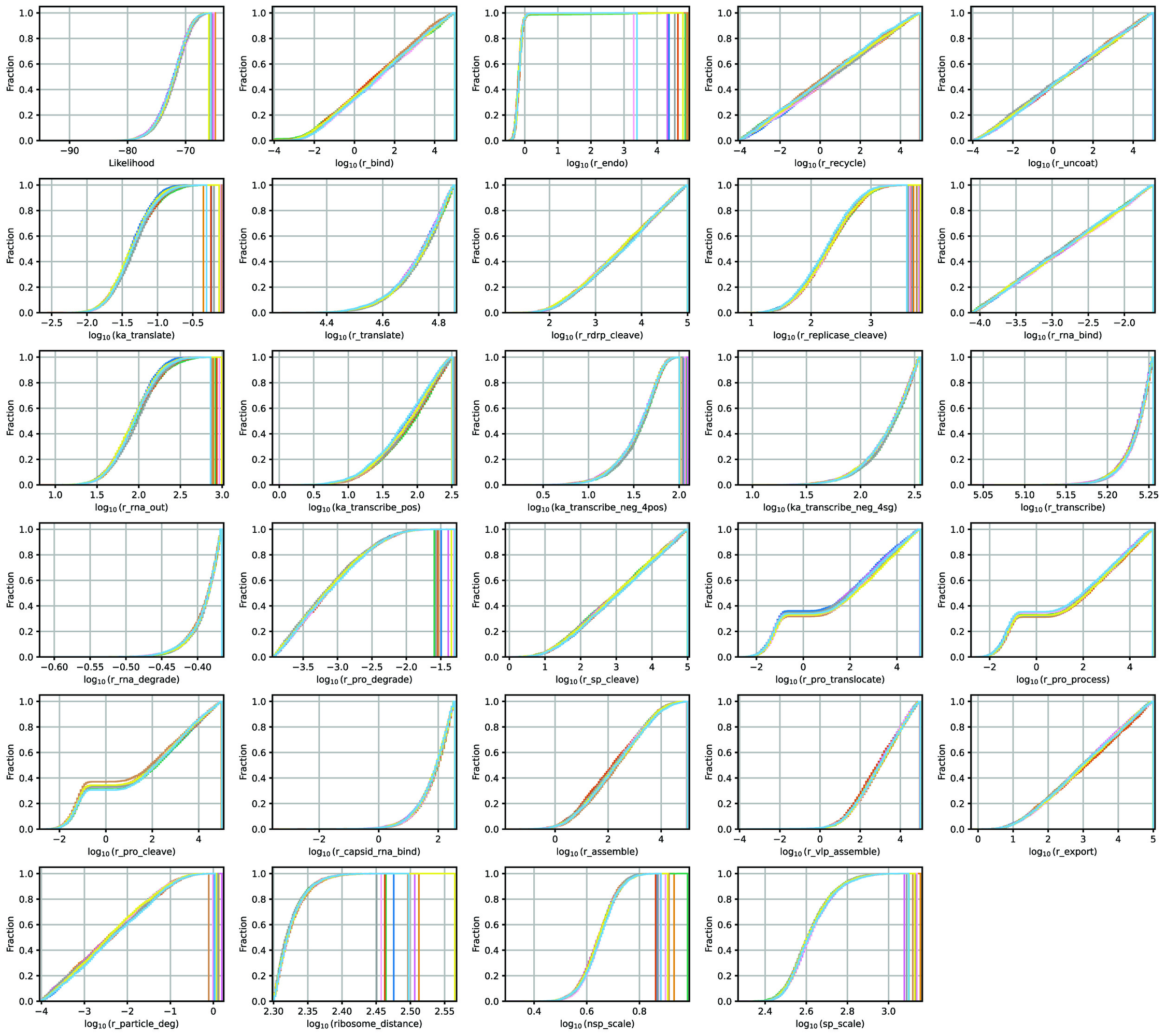

Supplement: S3 Fig — Model calibration to the RNA genome strand ratio constraint and experimental data was run 10 times given different initial configurations. The top left plot corresponds to the likelihood distributions (see Eq 11). The remaining 28 panels correspond to the 26 estimated model parameters and 2 scaling factors. (TIF) [file pcbi.1013082.s005.tif]

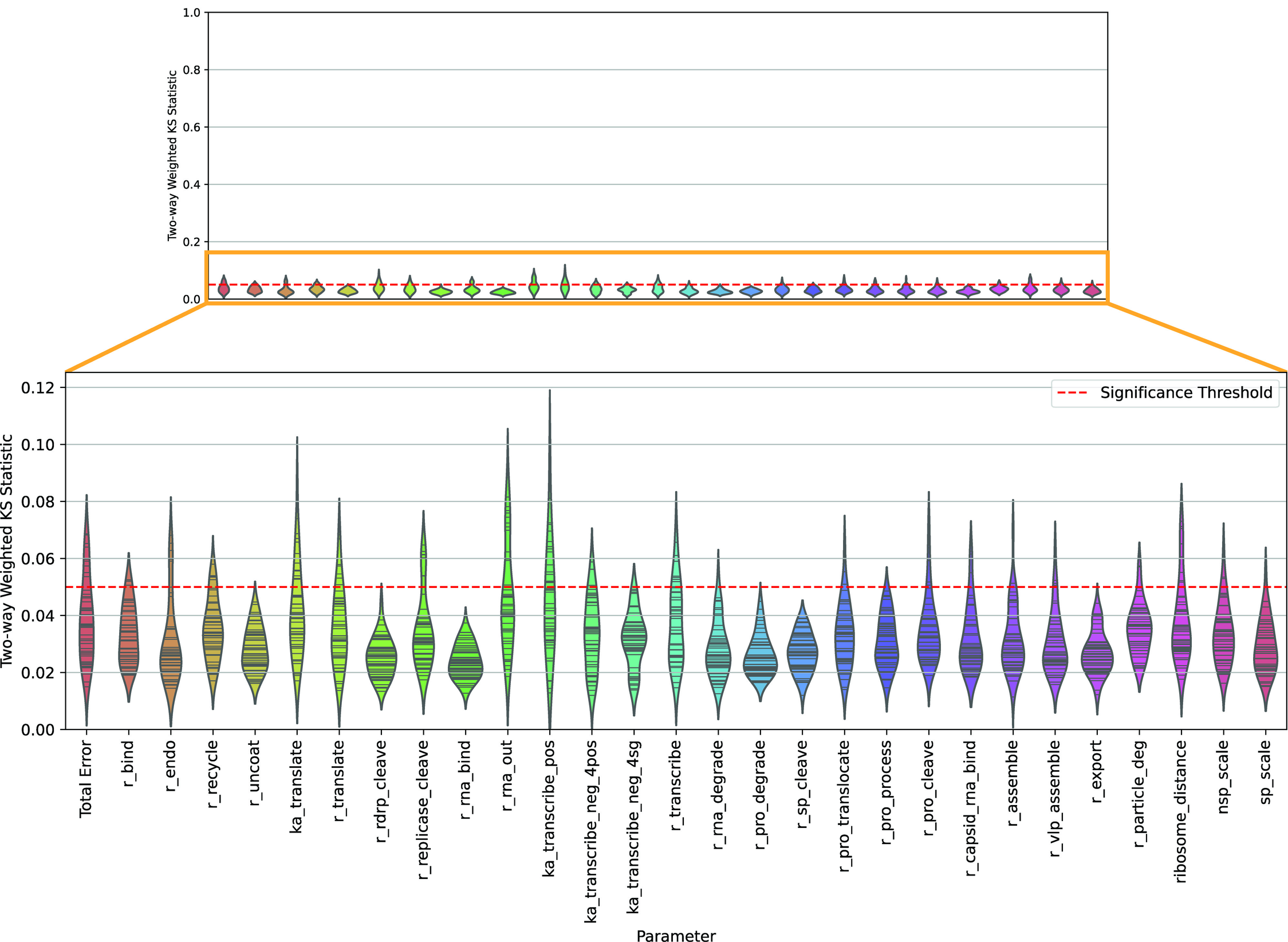

Supplement: S4 Fig — Using the resulting marginal cumulative distributions generated by each run of model calibration, as shown in S3 Fig, the two-way weighted Komolgorov-Smirnov statistic between each combination of runs (10 runs, 45 pairs for comparison) for the likelihood (labeled Total Error and defined in Eq 11), 26 model parameters, and 2 scaling factors. The black lines in each violin plot represent individual KS statistic for a pair of runs. (TIF) [file pcbi.1013082.s006.tif]

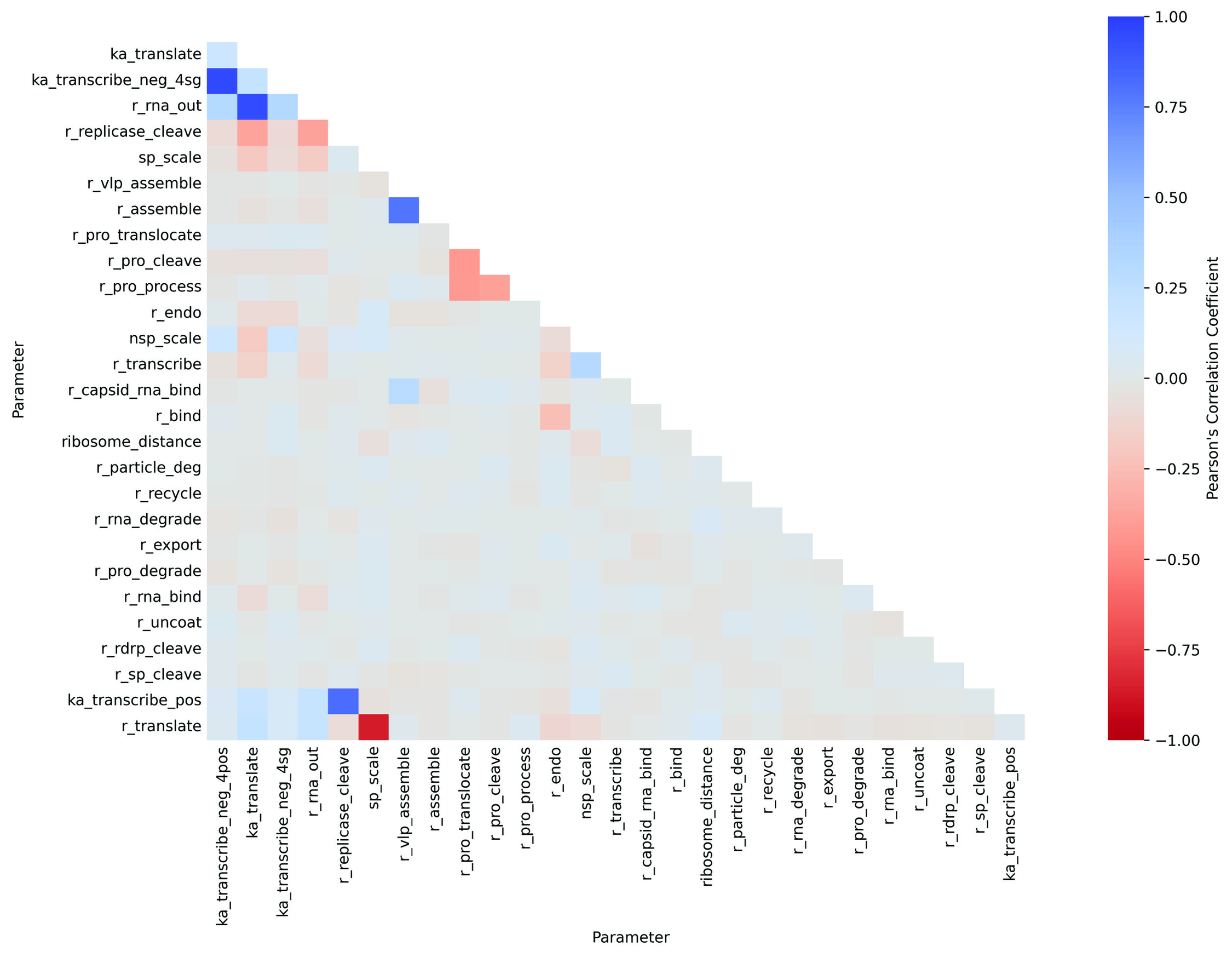

Supplement: S5 Fig — The Pearson correlation coefficients between calibrated parameters (n = 28, 26 model parameters and 2 scaling factors) are calculated from the parameter set ensemble (n = 2,500). (TIF) [file pcbi.1013082.s007.tif]

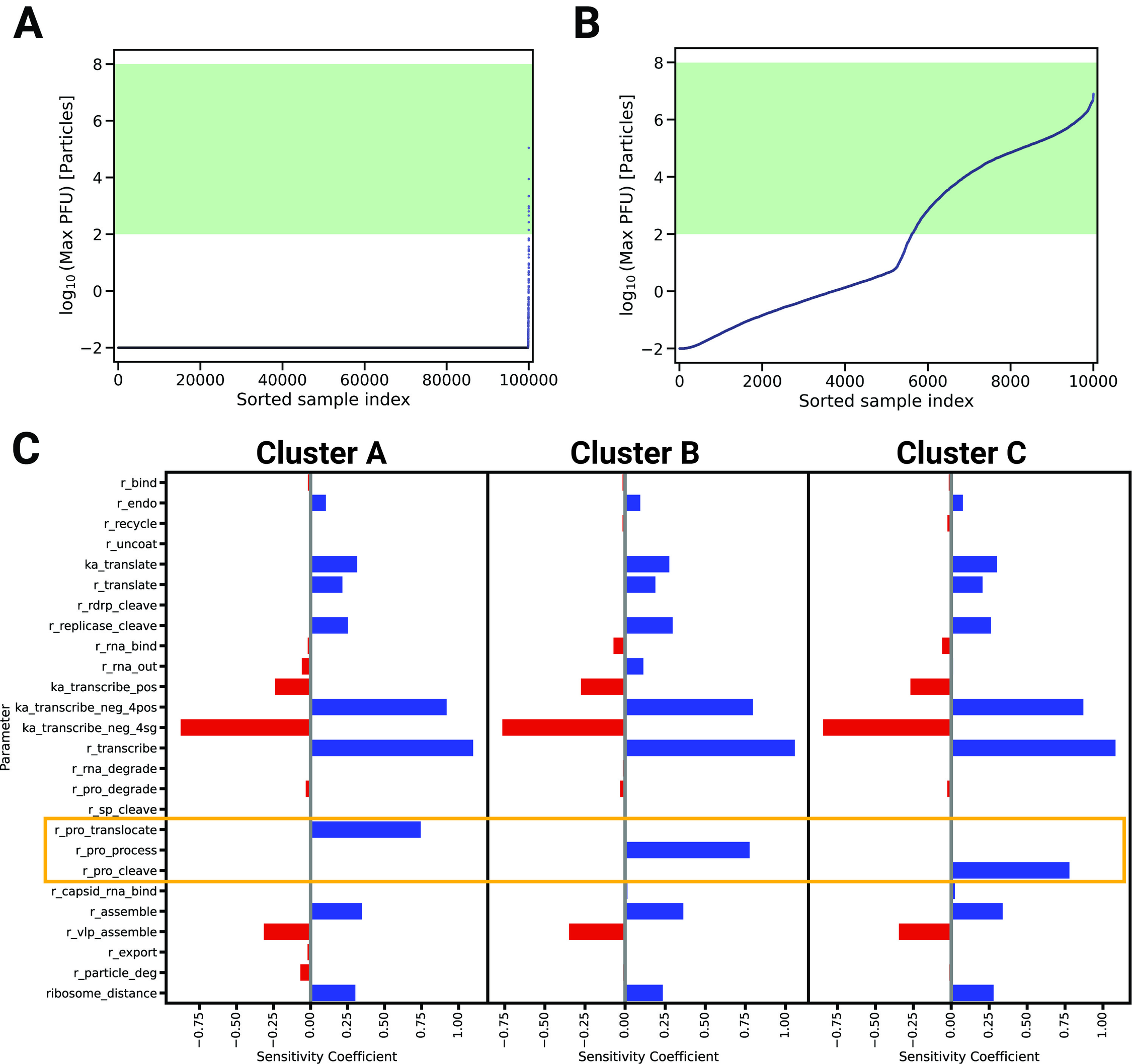

Supplement: S6 Fig — (A) Sorted distribution of maximal PFU yield from parameter set samples generated from global sampling with calibration bounds specified in S1 Table. (B) Sorted distribution of maximal PFU yields of parameter set samples generated using the best posterior set sample as described in Materials and methods. The number of parameter set samples generated are 105 and 104 for panels A and B, respectively. Green shaded regions in panels A and B correspond to the filtered PFU count range for samples to be used for the linear regression. (C) Cluster-based linear regression sensitivity analysis reveals three modes of post-translational processing dynamics. The sensitivity coefficients when sampling around the best posterior parameter set from each individual cluster (n = 3) using the same method described in Fig 7A. As shown in Fig 6, the left plot corresponds to the cluster in which r_pro_translocate is the rate-limiting parameter. The center shows the resulting sensitivity coefficients when sampling parameters around the best sample cluster in which r_pro_process is rate-limiting. The right plot corresponding to the sensitivity coefficients for model parameters using the best parameter set from the cluster in which r_pro_cleave is rate-limiting. (TIF) [file pcbi.1013082.s008.tif]
