# Supplementary material for: A detailed kinetic model of Eastern equine encephalitis virus replication in a susceptible host cell
Source: PLoS Comput Biol. 2025 Jun 4;21(6):e1013082. doi: 10.1371/journal.pcbi.1013082 (PMC12136344; doi:10.1371/journal.pcbi.1013082)
Supplement: Attachment — Submitted filename: Response to Reviewers.pdf [file pcbi.1013082.s011.pdf]

Reply to the Reviewers

Re: Manuscript ID PCOMPBIOL-D-24-02237

"A detailed kinetic model of Eastern equine encephalitis virus replication in a susceptible host cell"

Caroline I. Larkin, et al.

PLOS Computational Biology

March 27, 2025

Dear Drs. Antia and Wodarz:

We would like to thank you and the reviewers for the time and effort you have dedicated to handling and reviewing our reviewing our manuscript. We are grateful for your constructive feedback, which has helped us improve the clarity, rigor, and overall quality of our work. We are hopeful that incorporating these suggestions has greatly improved the presentation of our work and that the reviewers will now find it appropriate for publication in *PLOS Computational Biology*.

Sincerely,
James R. Faeder, PhD
Associate Professor
Department of Computational and Systems Biology
University of Pittsburgh

Summary of changes

Major changes:

- The code used to generate the data presented in this manuscript is now available at `https://github.com/LarkinIt/EEEV-Replication-Model`
- All data generated by the code for the manuscript is available on Zenodo: `https://doi.org/10.5281/zenodo.15080538`.
- We have expanded Figure S6 to include the distribution of viral production when using a global sampling scheme (Figure S6A) and the sampling scheme shown in Figure 7 (Figure S6B). The data used to generate this file is now included in the Zenodo repository.
- Text that has been changed or added in the manuscript to address reviewer comments appears in red for the convenience of the reviewers.

Pointwise responses to feedback

Reviewer #1, comment #1

Overall this manuscript was well-written and easy to understand with clear conclusions from the modeling efforts. I find this work to be potentially impactful and to be nearly ready to publish. I have a number of minor typographical suggestions and just two minor questions/concerns to address to further polish the manuscript. I have no major concerns.

Our response #1.1

We thank the reviewer for this positive assessment of our work.

Reviewer #1, comment #2

The use of weights from the linear regression as the sensitivity coefficients is the least clear part of the manuscript. In the methods, a sample equation would aid in clarity to ensure that others could reproduce this technique. Alternatively, the equation could be inserted at line 386. I've seen a lot of linear regression and a lot of local and global sensitivity analyses methods, but I am not familiar with this approach of combining the two.

Our response #1.2

We thank the reviewer for pointing out the lack of clarity on the sensitivity analysis method, which is simple but not commonly used. As the reviewer has suggested, we modified description of the method in the Results section starting at line 389 to clarify the correspondence between the regression coefficients and the sensitivities as follows:

> We then performed linear regression to approximate the PFU production (y) as a linear function of the log parameter values ($\vec{x}$),
>
> $$y(\vec{x}) = c_0 + \sum_{i=1}^{N} c_i x_i + \varepsilon, \tag{1}$$
>
> where each coefficient c_i, estimated by linear regression, represents the linear sensitivity of the output y with respect to the feature x_i, and ε is the error.

A reference to this equation has also been placed in the Methods section at line 863.

Reviewer #1, comment #3

In the text, the references included in Table S1 are mentioned. Are these numerous sources cited in the main text as well? If only in the supplemental spreadsheet, then the sources won't be linked to your paper online and the authors of the work that you used to support aspects of your work won't get increases to their citations through the online indexing services. My suggestion is to make sure that everything you've cited in your supplemental materials is also cited somewhere in the main text.

Our response #1.3

We thank the reviewer for raising this important point. We have now cited all of the references in Table S1 in the main text at lines 626 and 793.

Reviewer #1, comment #4

Suggestions for improving text:

- *In the 2nd sentence of the author summary, "in humans" is used twice.*

- *Sometimes inconsistent use of the Oxford comma. E.g., line 362 after B (orange) is missing a comma and Fig 5 caption after (TC:sg).*
- *Lines 113-119 contain one continuous long sentence. While it is grammatically correct, it is recommended to break into two sentences starting from the clause that starts with "which undergoes..." at line 114.*
- *Line 222: insert "with" after "consistent".*
- *Line 226: no period at the end of the heading.*
- *Line 229: redundant word "that that".*
- *Line 285: insert comma after "polyproteins".*
- *Line 300: insert comma before "respectively".*
- *Line 301: insert comma after "depletion".*
- *Fig 4 caption: Remove (B) before "Parameter scans" and "in panel B" after "red line".*
- *Fig 5 caption: no space in 2,500.*
- *In the Fig 7 caption, parenthesis around the numbered steps would be clearer than the periods that occur midsentences now. E.g., "viral production, 1) 10,000 parameter setps are generated and 2) subsequently simulated."*
- *Line 499: the text that follows the colon for the two-pronged approach includes three items (shutdown, translation, recruitment) and can be read with different interpretations. So it'd be helpful to be explicit to readers about how you want these grouped by including numbered items. I think you meant "two-pronged approach: (1) shut down of host transcription and translation and (2) recruitment"*
- *In the equation/unit conversion that follows line 788, there is an unnecessary ratio of 1 mol / 1 mol.*
- *Line 764: 28 parameters are listed here. To aid in clarify, I suggest including as you did in the Fig S5 caption that there are 26 model parameters and 2 scaling factors.*
- *Line 789: paragraph should not be indented.*
- *Line 790: space between the number of the volume units. Also mu shouldn't be italicized. (If using latex, this can be accomplished with* `\upmu` *in the upgreek package.)*
- *Line 808: i.e. should be followed by a comma*
- *Line 824: no space in the number 10,000.*
- *Line 841: cells doesn't need to be capitalized.*
- *Line 842: comma after kidney is not needed before defining the acronym (BHK).*
- *Line 879: Is capital T = 0 time? If so, lowercase t is used earlier, so it'd be clearer to keep the notation consistent.*

- *Several instances of starting a decimal number with the decimal instead of having a leading 0 before the decimal, throughout the methods. E.g., on line 896, .2 mL instead of the more standard 0.2 mL with the leading 0.*
- *The RT-qPCR assay section and following in the methods doesn't have any indentation, while earlier section indent paragraphs after the first.*
- *S2 Fig caption: Insert comma before respectively.*
- *S5 Fig caption: Remove space in 2,500.*

Our response #1.4

We thank the reviewer for pointing out these typographical errors and other issues, which we have now corrected.

Reviewer #1, comment #5

When acronyms are defined or viruses named, only the proper nouns need to be capitalized. This is correct in some places (abstract), but incorrect in several other instances throughout the manuscript for Eastern equine encephalitis, hepatitis C, Venezuelan equine encephalitis, Latin hypercube sampling, Markov chain Monte Carlo, preconditioned Monte Carlo, sequential Monte Carlo with normalizing flows.

Our response #1.5

We thank the reviewer for catching these issues. We have now edited the entire manuscript to be consistent with capitalization. In particular, we refer to the capitalization used in the original paper presenting preconditioned Monte Carlo as a guide for formatting preconditioned Monte Carlo, Sequential Monte Carlo, and Normalizing Flows.[2]

Reviewer #1, comment #6

Fig 4C: I understand that the probability density has the same x-axis as the diagram, but it doesn't share either of the two labeled y-axes. That makes this panel a bit confusing. Perhaps just having the density on a separate panel as you did in e.g., Fig 6 D-F would be clearer.

Our response #1.6

We thank the reviewer for pointing out this issue. We have amended Figure 4C to now plot the probability density of `ribosome_distance` above the original panel so that it shares an x-axis with the original plot but contains its own y-axis.

Reviewer #1, comment #7

Fig 6 is overall very clear. However, panel B is less clear than the rest. Could the cluster labels be added to the groups in panel B?

Our response #1.7

We thank the reviewer for the positive comment. We have now updated Figure 6 to include the cluster labels in panel B.

Reviewer #1, comment #8

S1 Fig has 29 panels for 28 distributions. What is in the first panel? I can't easily find what log(C) is.

Our response #1.8

We thank the reviewer for catching this potential point of confusion. We have now amended S1 Fig to use the same notation as the manuscript. In particular, the figure caption now mentions that the top left panel in S1 Fig corresponds to the likelihood function, which is defined in Equation 10.

Reviewer #1, comment #9

S5 fig and S6 fig are not cross-referenced in the text.

Our response #1.9

We thank the reviewer for highlighting this missing information and apologize for our oversight. We have now included references to Figures S5 and S6 in Lines 224, 256, 287 and Lines 385, 389, 417–418, respectively.

Reviewer #2, comment #1

This paper presents a computational analysis of Eastern Equine Encephalitis Virus (EEEV), focusing on modeling its intracellular replication. The authors use a rule-based model describing the live cycle of EEEV from attachment to export of virus (and VLP) in mammalian cells. Bayesian inference was used to calibrate the model and reduce uncertainty. The model describes the experimental data well and predicts the important role of ribosomes in viral and production and the EEEV life cycle in general. The study addresses an important topic in virology given EEEVs high mortality rate and the lack of available treatment options. The paper is well written and presents an interesting approach for such a complex model. Does the cell line (BHK-21) you use have any immune response? If yes, please explain why the immune response does not affect the viral dynamics? If no, please mention that.

Our response #2.1

We thank the reviewer for the positive feedback and making this important point about the cell culture. The BHK-21 cell line is defective in interferon production.[3] We have now mentioned this in the manuscript in Lines 142 and 871–872.

Reviewer #2, comment #2

One model assumption is that ribosomes are within replication spherule? Are there any references?

Our response #2.2

We thank the reviewer for the interesting question. From our understanding, the exact location of host ribosomes used for viral replication is relatively understudied. However, studies have shown that translation to produce the nonstructural proteins is required for the formation of replication spherules.[1] Given the ambiguity of the precise location of host ribosomes, the model does not distinguish host ribosomes based on their presence within the RS.

Reviewer #2, comment #3

In Table S1 (rate rules) maybe add a column with the name of the step/process.

Our response #2.3

We thank the reviewer for the helpful suggestion. We have now included an additional column in the Rate Rules tab of Table S1 called `Process` for the convenience of the readers.

Reviewer #2, comment #4

How many parameters are estimated? 26 or 28 (including two scaling factors). You use both numbers (e.g., in lines 154, 541, 764).

Our response #2.4

We thank the reviewer for drawing our attention to this discrepancy. To clarify, there are 26 unknown model parameters and 2 scaling factors used to scale the model outputs to the experimental data. Thus, there is a total of 28 parameters that are estimated during model calibration. To make it clear, we have edited Lines 214 and 771, the Fig S3 caption and the Fig S4 caption to distinguish the quantity of model parameters and scaling factors.

Reviewer #2, comment #5

Can you say anything to the estimated values or are model predictions using Bayesian inference entirely qualitative?

Our response #2.5

We thank the reviewer for bringing up this important point. The ability of the model to make quantitative predictions related to model parameters and observables is closely related to the issue of parameter identifiability, discussed in the section "Calibration of model parameters with Bayesian parameter estimation" in the paragraph starting at line 209. We have modified the first two sentences of this paragraph to read

> A notable benefit to Bayesian inference methods is the ability to quantify the uncertainty of model parameter values, often referred to as parameter identifiability, and model predictions using the posterior distributions of the estimated parameters, as we have already shown in Fig 2. The narrower the posterior distribution of a model parameter, the more identifiable it is.

We see that using the combination of the experimental data and the RNA genome strand ratio constraint, about 14 out of the 28 model parameters are constrained to a range of less than three orders of magnitude, and "several biologically important and potentially observable parameters including `r_endo`, `r_replicase_cleave`, and `ribosome_distance`, become well-constrained" (lines 223–224). In addition, as we discuss in the text, the model makes reasonably well-constrained and testable predictions about such observable quantities such as the particle-to-PFU ratio (Fig 2B), subgenome-genome promoter affinity ratio (Fig 2C,D), and the translocation-translation ratio for viral RNA (Fig 3G,H). Overall, the use of Bayesian inference is able to provide quantitative, testable estimates of a significant number of features of EEEV infection dynamics.

Reviewer #2, comment #6

- *In the author summary, second sentence: you wrote twice "in humans"*
- *line 477: double "by setting by setting"*
- *line 707 and 801: in those sentences may be a word missing*

Our response #2.6

We thank the reviewer for pointing out these typographical errors, which we corrected.

Reviewer #3, comment #1

The authors present a very comprehensive and well-written manuscript describing a mathematical model of the intracellular replication of eastern equine encephalitis virus, which they have calibrated to time-resolved experimental data of the infection kinetics measured in BHK-21 cells. The authors provide a detailed description of their model and the assumptions they make. The model is a rather large model with 36 equations and a similar number of model parameters, raising some questions about model identifiability. Parameter estimation is done using a Bayesian sampling approach, which provides not point estimates, but posterior distributions over model parameters. The model analyses done include a sensitivity analysis and an identifiability analysis based on points sampled from the posterior distribution; sensitivity analysis results reflect essentially findings in other models developed for related (and simpler) RNA viruses such as HCV or Dengue virus. This paper presents the first model for (the more complex) alpha-viruses.

The work presented is very solid work. I do have only a minor questions concerning the MCMC sampling approach used for parameter estimation. The preconditioned monte carlo approach with normalizing flows used by the authors is not well known in computational biology, and i suggest to add some more details. Of importance, I assume this MCMC-based algorithm requires a burn-in phase to ensure the Markov Chain reaches its stationary distribution, or to ensure proper learning of the normalizing flows. Can the authors comment? A limited analysis is done with different starting points; I believe this point is critical though to ensure convergence of the sampler to the model posterior distribution. Furthermore, the authors appear to use only default values for the parameters of the preconditioned monte carlo algorithm. Can the authors be sure the 2500 points they sample are sufficient to get a reasonable sample from the posterior?

Our response #3.1

We thank the reviewer for the positive feedback on the work presented in our manuscript. Regarding the Bayesian inference method used in this manuscript, Sequential Monte Carlo (SMC), and thus, preconditioned Monte Carlo (PMC), are particle-filter-based methods. Unlike Metropolis-Hastings or Parallel tempering MCMC, SMC infers distributions by iteratively sampling and evolving an ensemble of particles/samples. We have added additional detail about the PMC method to our methods section starting at Line 808.

With respect to the number of samples, we followed the original documentation guidelines for the PMC python package `pocoMC` (`https://pocomc.readthedocs.io/en/latest/sampling.html`), which are as follows:

> We recommend values around `500` for most problems and increasing that to `2000-4000` particularly challenging distributions.

References

[1] Zeinab Elmasri, Benjamin L. Nasal, and Joyce Jose. Alphavirus-Induced Membrane Rearrangements during Replication, Assembly, and Budding. *Pathogens*, 10(8):984, August 2021.

[2] Minas Karamanis, Florian Beutler, John A Peacock, David Nabergoj, and Uroš Seljak. Accelerating astronomical and cosmological inference with preconditioned Monte Carlo. *Monthly Notices of the Royal Astronomical Society*, 516(2):1644–1653, October 2022.

[3] K. Otsuki, J. Maeda, H. Yamamoto, and M. Tsubokura. Studies on avian infectious bronchitis virus (IBV). *Archives of Virology*, 60(3):249–255, 1979.
